# Feasibility of a Nephrology Faculty Peer Observation of Teaching Pilot Program

**DOI:** 10.34067/KID.0000000000000138

**Published:** 2023-05-08

**Authors:** Jeffrey H. William, Stewart H. Lecker, Robert A. Cohen

**Affiliations:** Division of Nephrology, Department of Medicine, Beth Israel Deaconess Medical Center and Harvard Medical School, Boston, Massachusetts

**Keywords:** kidney, nephrology

## Abstract

- Awareness of being observed led to enhanced faculty preparation of teaching sessions.
- Any discomfort about being observed was generally outweighed by receiving observer input.
- Variable quality of feedback given to observed suggests need for more faculty development with next iteration of peer observation.

## Introduction

Nephrology has been changing rapidly in recent years, from the emergence of new medications to treat chronic kidney disease to advances in understanding the genetic basis of many kidney disorders. Consequently, such developments obligate alterations in the core curriculum taught to nephrology fellowship trainees. In our experience, medical educators are inclined to deliver the same presentations each academic year if no meaningful feedback is provided about the session. Novel educational approaches for teaching in the small group setting have been introduced in recent years that could be incorporated in teaching sessions to meaningfully engage learners.^1^ To assist our nephrology fellowship educators in improving on curriculum sessions, we piloted a peer-to-peer observation program designed to provide feedback about the learning environment, learner engagement, session management, and teaching methods. This program was based on a peer observation model that has proved successful in other medical education venues but has not been reported for use in medical subspecialties such as nephrology.^2–4^ In this study, we describe the pilot peer observation program and results.

## Methods

**Figure 1:**
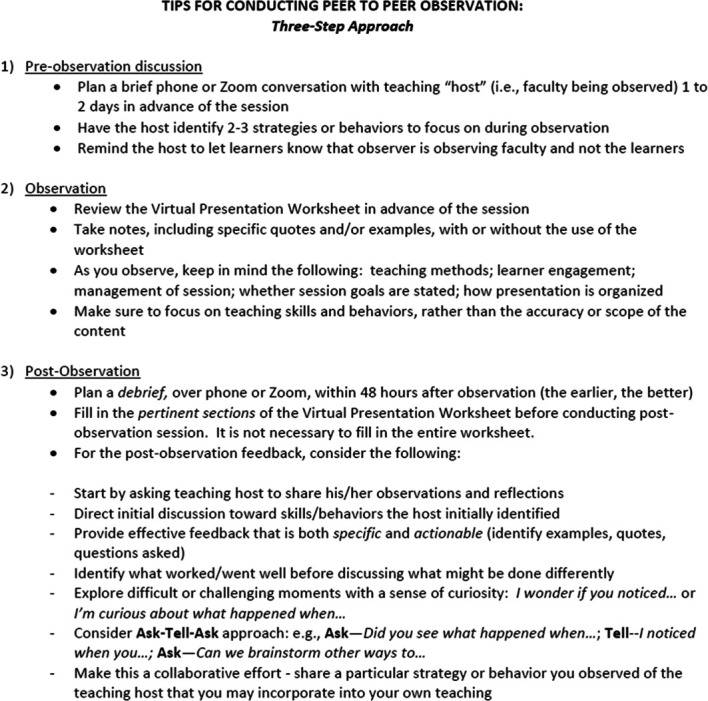
Peer observation guide.

Peer observations were scheduled based on the availability of five core clinician–educator faculty within the Division of Nephrology. The curriculum sessions occur twice weekly from September through June in the academic year. Each core faculty member was provided with a standardized rubric for small group teaching from a published resource to make sure all agreed on the format and the scope.^5^ We also provided a peer observation guide to all faculty observers and those observed to ensure consistency, transparency, and best practices (Figure 1). Each of the observing faculty members were instructed to reach out to the assigned session facilitator in advance of the scheduled session to obtain consent for peer observation, review the observation rubric, and address any particular aspects of the session for which feedback would be most appreciated. Owing to the coronavirus disease 2019 (COVID-19) pandemic, each session facilitator was given the option of delivering the talk through video conference or in-person.

As per the peer observation guide and standardized rubric, each observer was instructed to remain a pure observer (without involvement in the session itself). The fellows attending the session were informed of the observer's presence at the start of the session. After filling out the observation rubric, the peer observer could deliver the feedback to the session facilitator immediately after the session or within 48 hours of its completion.

At the end of the academic year, all observed teaching faculty were invited to complete a survey and participate in a focus group to discuss the experience in more detail.

(Survey can be viewed here—https://forms.gle/86AiJWNmhKtKT8hM9).

## Results

Data from the focus group session facilitated by an experienced non-nephrology educator are organized in Table 1. Three independent coders (J.H.W., S.H.L., and R.A.C.) evaluated the meeting transcript and agreed on the codes used to best describe the data. Based on code frequency and general review of the transcript, overarching themes emerged from the qualitative data, including observer effect, session preparation, presession and postsession meetings, and teaching faculty role in curriculum assessment. Subthemes and supporting quotations from focus group attendees are demonstrated in Table 1.

**Table 1:** Data from focus group of observed faculty

Overarching Theme	Most Commonly Referenced Subthemes	Quotations
Observer effect (experience of being observed)	Session modality/types of observation (video conference versus in-person)	“I'm trying to imagine how I would know whether fellows or the audience was engaged. If I was watching all the little squares on the screen and trying to figure it out.” “It's certainly easier for the observer to observe on Zoom, in a sense, because you can sort of be separate in a very real way.”
	Positive experience	“We know each other very well and I think care for each other very deeply. It's a very safe community.” “…didn't feel like (peer faculty presence) was making me nervous, but I definitely feel like there was somebody else there.”
	Supportive peer colleagues	“I think it's really different being not only a peer, but a colleague that's observing you and giving you feedback that…you know, they know the fellows too. They know your audience because they've done it. And they're giving you feedback that you know you can trust.” “…we're doing this for the benefit of each other.”
	Motivation to improve performance	“I think there's nothing more stimulating for preparation or just thinking about the talk when you know you're going to be observed by one of your peers.” “…make sure I had the right studies and the right data and I was showing it correctly. So I was more concerned about the factual information and making sure I had it right.”
Session preparation	Updating and revisions	“I don't always make huge changes from year to year.”
	More interactive/increased engagement	“…more about the organization and engagement rather than the content.”
Presession and postsession meetings	Types of feedback requested	“…wanted feedback…on how interactive it was and how much I was engaging the fellows” “…whether I was keeping the fellows engaged and whether the amount of material...was appropriate or…ideas of how to manage content.
	Feedback delivery	“I think we're not as good at giving (feedback) as we could be.” “I think we all need to learn a little bit more about giving feedback to each other and how to do it in a way that is helpful to our peers.”
Teaching faculty role in curriculum assessment	Iterative observations	“I think I'd like to see it back and forth…like this is the culture of the place. And this is the culture of how we do our didactics and that by over the years, you get to a better place.”
	Sources of feedback	“I WANT somebody to watch me actually do [the teaching], to tell me that it’s for real.”

**Figure 2:**
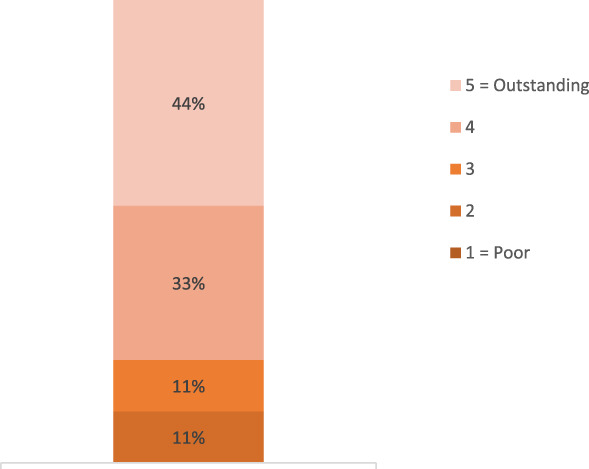
**Overall rating of peer feedback program.** Survey respondents (*n*=9 of 17 educators) were asked to provide their overall rating of the peer feedback program on a Likert scale of 1–5, with 1=poor and 5=outstanding.

Of 17 educators within the curriculum, nine responded to a program evaluation survey (53% response rate). The vast majority of these respondents facilitated at least two sessions per academic year, with just over half having been observed in a teaching role in the past. In total, 33 teaching sessions were observed during the academic year, divided among five observers (4–8 sessions per observer). More than half of the respondents indicated that they prepared differently knowing they would be observed and nearly half changed some aspect of their session about receiving feedback (Supplemental Figures 2 and 3). Overall, the peer feedback program was well received among the faculty responding to the survey (Figure 2).

## Discussion

A peer observation pilot program to provide feedback to nephrology fellowship educators was both feasible and effective. Educators involved in this effort appreciated the spirit of collegiality and continuous quality improvement of curriculum content. These findings are consistent with previous studies in the field of peer feedback that have shown both observers and observed value the process and find the exercise useful.^6^

While those observed did experience some stress in this setting, the minimal discomfort seemed to be outweighed by the benefit of the feedback received. An initial concern in the design of this program was that the presence of the observer would fundamentally change the teaching session in negative ways, both for the facilitator and for the learners. However, our findings are consistent with previous work that has shown the presence of peer observers to be a positive influence on the session.^7^ Multiple educators in our program cited increased motivation to improve the session on knowing they would be observed. Educators spent extra time in preparation, with an enhanced focus on content accuracy and delivery, fellow engagement, and an interactive and safe learning environment. It was also agreed that mutual trust and collaboration between observers and those observed was a key aspect of a successful peer feedback program, where the positive intent of the feedback was duly recognized.

Because this pilot program was rolled out in the midst of the COVID-19 pandemic, many curriculum sessions were converted to a video conference format (Zoom Video Communications, Inc., San Jose, CA) (see Supplemental Figure 1). Although there was some initial concern about whether the feedback would be applicable to one of these sessions, it has become increasingly clear that the video conference format is both convenient and effective when used thoughtfully. Observers noted that it was much easier to blend in compared with an in-person session, as muting the camera and microphone rendered the peer observer present but not seen or heard. There has been extensive research and recommendations regarding synchronous online learning and peer feedback in a variety of contexts,^8–10^ but not within the curricula of medical subspecialty fellowship training programs.

Our program also served as a faculty development opportunity, as required by the Accreditation Council for Graduate Medical Education (ACGME) Common Program Requirements for fellowships,^11^ guiding the creation of engaging teaching sessions through peer feedback. Focus group data also revealed additional opportunities for faculty development, including training in delivering peer feedback. While nearly all the peer observers and faculty observed had a favorable opinion about the peer feedback program generally, special mention was made about the variable ability of faculty observers to provide meaningful feedback in an effective manner. The desire to continue moving this program forward is contingent on training faculty to both give and receive feedback skillfully in this unique setting, as evidenced in other higher education learning environments as well.^12,13^

We believe that future iterations of this faculty peer feedback program would be improved with additional efforts in increasing faculty comfort with the idea of peer observation and feedback. A more robust introductory meeting before any observations would be helpful in setting expectations of the presession and postsession feedback and getting buy-in from skeptical faculty. Within this session, we would include an open discussion among faculty balancing the expected initial discomfort of being observed with the overall benefits of receiving feedback to become a better educator and improve the curriculum as a whole.

Faculty peer observation of teaching may serve to enrich medical subspecialty fellowship curricula in nephrology, by improving both quality of teaching and content. Our experience through implementing this program for the full academic year shows that this educational effort is not only feasible but nearly universally appreciated by the involved faculty and likely to provide the highest-quality educational experience for fellows.

## Supplementary Material

SUPPLEMENTARY MATERIAL

## Supplemental Material

Supplemental Figure 1. Modality of observed sessions.

Supplemental Figure 2. Presession feedback.

Supplemental Figure 3. Postsession feedback.
