# Supplementary material for: Feasibility of a Nephrology Faculty Peer Observation of Teaching Pilot Program
Source: Kidney360. 2023 May 8;4(6):e824–7. doi: 10.34067/KID.0000000000000138 (PMC10371357; doi:10.34067/KID.0000000000000138)
Supplement: SUPPLEMENTARY MATERIAL [file kidney360-4-e824-s001.pdf]

Supplementary figures

Supplementary Fig 1 – Modality of observed sessions

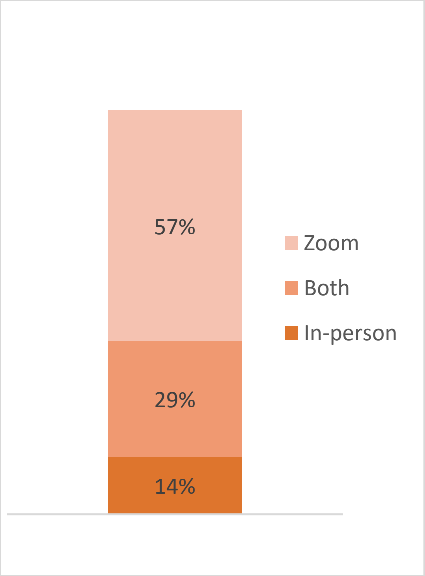

Suppl Fig 1 caption – Respondents were asked to indicate whether their observed sessions took place in-person or over video conference (Zoom). Multiple educators teach more than one session and were instructed to indicate "Both" if they used each modality for different sessions over the academic year.

Supplementary Fig 2 – Pre-session feedback

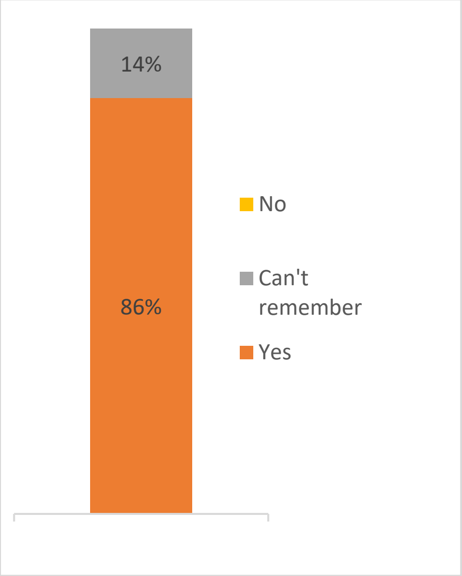

Suppl Fig 2 caption: Respondents were asked "Did the peer observer ask you what you wanted feedback on in advance of the upcoming session?" Answer choices included Yes, No, and Can't remember. 86% of survey respondents attended a pre-session meeting.

Supplementary Fig 3 – Post-session feedback

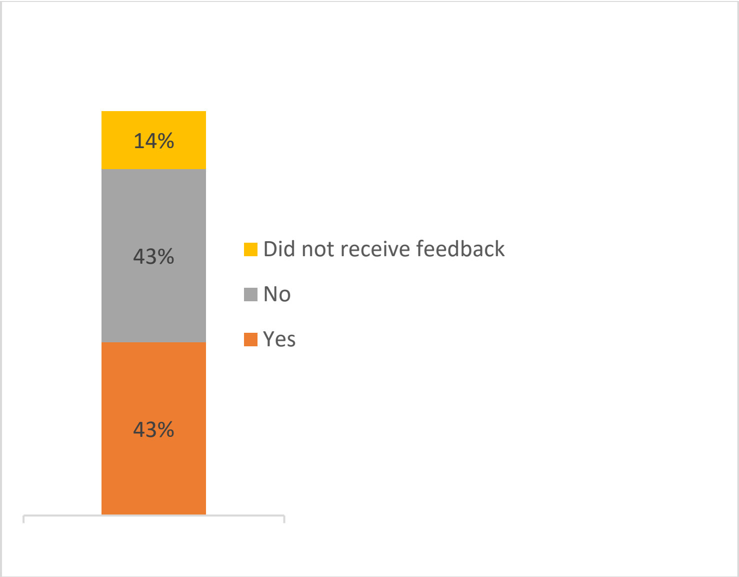

Suppl Fig 3 caption: Respondents were asked “Did you change your session(s) in response to the feedback you received?” Answer choices included Yes, No, and Other (with response of “Did not receive feedback. 86% of survey respondents received post-session feedback.
